# Higher Onset of Periprosthetic Joint Infections in Patients With Teeth Compared to Those Without Teeth

**DOI:** 10.7759/cureus.63696

**Published:** 2024-07-02

**Authors:** Joseph D Brenner, Marina Atallah, David Yatsonsky, Andrew Casabianca, Maged Hanna

**Affiliations:** 1 College of Medicine and Life Sciences, The University of Toledo, Toledo, USA; 2 Orthopedic Surgery, The University of Toledo Medical Center, Toledo, USA; 3 Anesthesiology, ProMedica Toledo Hospital, Toledo, USA

**Keywords:** primary total hip arthroplasty, edentulous patients, primary total knee arthroplasty, oral microbiome, periprosthetic joint infection

## Abstract

Introduction: Bacteria can enter the bloodstream through simple actions such as brushing teeth, flossing, and even chewing food, increasing the chance of hematogenous seeding of prosthetic joints. Antibiotics before dental work in patients with orthopedic hardware is a topic of debate because of concerns for antibiotic resistance. Patients with dentures theoretically avoid this risk due to the lack of teeth and their maintenance. Most periprosthetic joint infections (PJIs) that occur in the first six months after surgery are due to wound infection, whereas late PJIs are more commonly caused by hematogenous seeding.

Materials and methods: Charts from patients who received primary total joint arthroplasty were interrogated for the condition of their teeth at the time of operation. If the patient had a PJI, the time from surgery and the organism responsible were noted. Multivariate linear regressions were performed for statistical analysis to compare rates of dental status, infection, comorbidities, and demographics.

Results: From the 1,500 charts reviewed, patients with teeth and edentulous had similar rates of comorbidities. PJI patients had higher rates of chronic kidney disease than patients who did not have the infection. The overall rate of infections in patients with teeth was 2.14%, close to the national average. The rate of infection in patients without teeth was 0.78%. Patients with teeth have a higher rate of infection one month or longer from surgery than edentulous patients.

Conclusions: There was an increased infection rate in patients with teeth at six months and greater since the primary total joint arthroplasty. The organisms responsible for many of the PJIs are commonly found in the mouth of humans. Having teeth is a potential risk factor for late PJI.

## Introduction

As the average life expectancy in the United States increases closer to 90, the number of total joint arthroplasties (TJAs) performed is expected to rise exponentially with it [1,2]. The primary procedures included in TJAs are total knee arthroplasties (TKAs) and total hip arthroplasties (THAs). One of the greatest complications associated with such a procedure is periprosthetic joint infection (PJI), occurring in up to 2% of cases, along with periprosthetic fracture, osteolysis, metallosis, etc. [3]. Bacteria can create a biofilm on the metal of the artificial joint that renders most antibiotics ineffective, requiring treatment for PJI in staged revision exchange arthroplasty (67%), irrigation and debridement (33%), or one-stage reimplantation (4%) [4]. Two-stage arthroplasty has a success between 65% and 90% depending on the joint and severity of infection, meaning fusion, resection, and arthroplasty are still performed under certain circumstances [5]. Patients with PJI not only endure additional invasive procedures, but also undertake great economic hardships as most spend over a week in the hospital and accrue a bill of nearly $100,000 [4,5]. To avoid PJI, practices such as prophylactic antibiotics are given to patients with TJA prior to dental appointments and clearance is often obtained from a dentist prior to the operation to ensure there are no dental carries that could lead to complications [6-8]. As the fear and incidence of antibiotic resistance rise and the lack of sufficient evidence supporting the link between oral infection and PJI has been brought into the spotlight; the guidelines concerning dental clearance and prophylactic antibiotics have recently been challenged [8,9]. The rate of bacteremia from everyday tasks such as chewing (17%), brushing (44%) and flossing (41%) is relatively high and the bacteria found most commonly in PJI are coagulase-negative *Staphylococcus*, *Staphylococcus aureus*, and *Streptococcus*, with occurrences of gram-negative species and *Enterococcus*, which can all be found in the oral cavity [4,6,7,10]. It stands to reason that if these organisms are in the oral cavity and the rate of bacteremia from the mouth is so high, then the rate of PJI should be much higher. However, studies have found that bacteria in up to 13% of PJI are commonly found in the oral cavity and that 11% of PJI stemmed from oral infections [6,11]. To further scrutinize the relationship between PJI and oral infection, we will compare the rates of PJI in patients who had dentures prior to their TJA and those with teeth. Our hypothesis is patients with dentures will have a lower incidence of PJI than those with teeth. Statistically significant different rates between these two groups will support this hypothesis and a lack of statistically significant data will refute it.

## Materials and methods

This was a retrospective chart review cohort study performed at the University of Toledo Medical Center and approved by the Institutional Review Board (IRB) of the University of Toledo in Toledo, USA. All necessary consent was obtained in a manner satisfactory to the IRB. All patients were at least 18 years of age and had undergone primary THA or primary TKA from January 1, 2010, and December 31, 2018, with at least three years of follow-up. Patients with a history of knee or hip revision surgeries, less than two years of follow-up, or major intraoperative complications such as fractures or excessive bleeding (>500mL) were excluded. Patients with morbid obesity with BMI >40.0 were also excluded. The study was conducted at the University of Toledo Medical Center Orthopedic Department and Anesthesia Department offices.

Our primary outcome was the prevalence of PJI in patients with dentition compared to patients who underwent the same surgery with dentures. The types of organisms causing PJI in patients with teeth and those with dentures were also compared to determine any discrepancies in potential susceptibilities. The data items that were collected include the date of surgery, the joint replaced, patient demographics (age, sex, race, BMI), comorbidities (diabetes mellitus, coronary artery disease, chronic kidney disease), and use of immunosuppressants. For cases that included PJI, the time between surgery and infection, the antibiotics given, and the organism responsible were also recorded. Procedures, interventions, and a Schedule A list of all primary joint replacement surgeries (hips and knees) were identified through computer search using the Current Procedural Terminology (CPT) codes for primary THA (CPT 27130) and primary TKA (CPT code 27447) performed at the University of Toledo Medical Center. The chart review process was conducted and de-identified. De-identified protected health information (PHI) had been collected. A master list was generated with identifiers and a code number throughout the research study. Statistical analysis was calculated using the Statistical Package for the Social Sciences (IBM SPSS Statistics for Windows, IBM Corp., Version 26.0, Armonk, NY) [12]. The number and percentage of patients in each experimental group were compared by demographic and comorbidities.

## Results

Of the 1,500 charts reviewed, 343 met one or more of the exclusion criteria. Of the remaining patients, 636 (55%) were female and 521 (45%) were male. The average age of the population was 67 years. TKAs were more common, comprising 706 (61%) of the cases while THAs filled out the remaining 451 (39%).

Collected data was then analyzed by two comparison groups: patients with PJIs and those who avoided them, and patients with teeth and those edentulous (Tables 1-2). After exclusions, 1,029 (89%) patients had teeth during their surgery and 128 (11%) did not. Patients with teeth and those edentulous had similar rates of comorbidities including smoking history, diabetes, blood loss during the procedure, coronary artery disease, use of immunosuppressants, and intraoperative complications such as iatrogenic fracture or neurovascular injury. Patients with PJI had a higher rate of chronic kidney disease (p = 0.03). Patients with teeth had a 2.14% rate of PJI compared to a 0.78% rate of PJI in edentulous patients (p = 0.30). Edentulous patients were an average of four years older at 69, compared to 65 for patients with teeth.

**Table 1:** The percentage of patients who underwent primary lower extremity total joint arthroplasty by infection status. The number and percentage of patients with infection compared by demographic and comorbidities of patients who avoid infection. EBL: estimated operative blood loss; CAD: coronary artery disease; CKD: chronic kidney disease

Characteristics	Infection, n (%)	Non-infection, n (%)
Teeth	22 (95.65)	1028 (90.74)
Edentulous	1 (4.35)	128 (11.29)
Obesity	14 (60.87)	650 (57.32)
Smoked	10 (43.48)	565 (49.82)
Diabetic	6 (26.09)	244 (21.52)
EBL ≥ 500mL	5 (21.74)	178 (15.7)
CAD	4 (17.39)	159 (14.02)
Autoimmune Disease	1 (4.35)	58 (5.11)
Immune Suppressed	2 (8.70)	90 (7.94)
CKD	5 (21.74)	78 (6.88)
Average Age	61	61
Female	10 (43.48)	624 (55.03)
Male	13 (56.53)	509 (44.97)
Knees	15 (65.22)	689 (60.76)
Hips	8 (34.78)	445 (39.24)
Total	23	1134

**Table 2:** A comparison of patients who underwent primary lower extremity total joint arthroplasty compared by tooth status. The number and percentage of patients in each experimental group were compared by demographic and comorbidities. EBL: estimated operative blood loss; CAD: coronary artery disease; CKD: chronic kidney disease

Characteristics	Teeth, n (%)	Edentulous, n (%)
Infections	22 (2.14)	1 (0.78)
Obesity	593 (57.63)	71 (55.47)
Smoked	507 (49.27)	68 (53.13)
Diabetic	221 (21.45)	29 (22.66)
EBL ≥ 500mL	160 (15.55)	232 (17.97)
CAD	139 (13.51)	24 (18.75)
Autoimmune Disease	52 (5.05)	7 (5.47)
Immune Suppressed	81 (7.88)	11 (8.59)
CKD	66 (6.41)	17 (13.28)
Average Age	61	63
Female	550 (53.45)	86 (67.19)
Male	479 (46.55)	42 (32.81)
Knees	617 (59.96)	82 (64.06)
Hips	412 (40.03)	46 (35.94)
Total	1029	128

PJI cases were on average 60 years old, while non-infected patients were 67. Of the PJIs, 13 (57%) were male and 10 (43%) were female and 432 (42%) of the non-infected patients were male and 597 (58%) were female. Both groups were two-thirds TKAs and the remaining third were THAs. Infected patients had a higher rate of chronic kidney disease (p = 0.04); however, the rest of the collected comorbidities appeared at similar rates.

**Figure 1:**
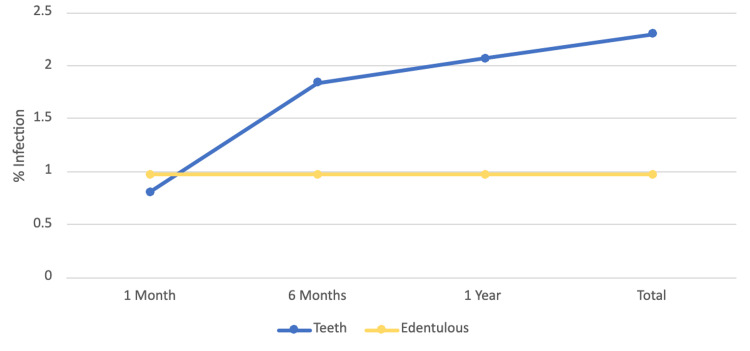
The percentage of primary lower extremity total joint arthroplasties infected by time from operation. The rate of infection from the time of operation in patients with teeth (blue line) and patients without teeth (yellow line) is based on the percentage of the cohort.

The single infection in the edentulous group occurred within one month of the procedure (Figure 1). Eight (35%) of the PJIs in patients with teeth happened within one month of the procedure, 18 (78%) had happened within the first six months, and 21 (91%) had taken place by the end of the first year. There was statistically significant data that patients with teeth had higher rates of infection after one month than those without teeth (p < 0.01).

Methicillin-resistant *S. aureus* (MRSA) was the most common infectious organism with nine cases, followed by polymicrobial infections at eight, methicillin-sensitive *S. aureus* (MSSA) at three, and *S. epidermidis* responsible for two PJIs. *Tuberculosis*, *Klebsiella*, *Corynebacterium*, and *S. constellatus *were all positive for one PJI each. Some of the infections were polymicrobial.

## Discussion

The goal of this retrospective chart review was to compare the rate of PJI in TKA and THA patients in patients with teeth versus those who were edentulous at the time of the procedure. Patients with teeth had an infection rate of 2.14%, which is similar to the national average of 2% [3]. Edentulous patients had an overall infection rate of 0.78%. Dentition status did not yield discrepancy within the first month of infection. However, there was a higher incidence of infection in patients with teeth in the late infection period after at least six months (p = 0.01). Our study was limited by the disparity in the size of the two groups. Additionally, the low prevalence of PJI means we expected few occurrences among the 128 edentulous patients. Even though there was only one PJI in that group, the expected value would have been only one to two more. Future studies should look for the rate of infection on a larger scale with experimental groups closer in size. Furthermore, other permanent orthopedic implants should be reviewed as well. Trauma surgeries with intramedullary rods and fixation should also be examined.

Out of the 128 patients who did not have teeth, the lone infection occurred in the first month due to *S. aureus*, most likely infecting the joint from the surgical site. Patients with teeth had a lower rate of infection at the one-month mark and had increased at six months, one year, and beyond. This could be due to the different bacteria and the route of infection from these bacteria through hematogenous seeding through the gums and teeth. Eight of the infections were polymicrobial which are common in dental caries such as *Streptococcus*, and one was caused by *Corynebacterium*, which is also commonly found in oral flora [13]. Additionally, since several were polymicrobial in origin, this could point to bacteremia from oral seeding. Six months after surgery, any bacteria that may have infected the joint from the wound show signs of infection, and the time period for diagnosis of acute infection has ended [14]. Patients with healthy immune systems who suffer infections after six months are generally infected via hematogenous spread [6]. Teeth cleaning and maintenance have a high rate of introduction of bacteria into the bloodstream, so teeth maintenance could provide a nidus for joint infection [15]. Sollecito et al. found no correlation between dental procedures and PJI [16]. Their study focused on dental procedures and did not consider daily brushing of teeth, flossing, or eating. This finding was repeated in Masuda [17]. Our study is retrospective and does not have the sample size to make recommendations on the use of prophylactic antibiotics for dental visits in the setting of TJA.

## Conclusions

Our data shows that patients with teeth have a higher rate of infection after the perioperative period, especially in the late PJI phase. This was true despite the comorbidities between the teeth and edentulous study groups, as well as between the infection and non-infection study groups. The bacteria found on pathology from the infections were commonly found in the oral cavity and several were polymicrobial. Our results, along with the observation of hematogenous seeding in late periprosthetic infections and the identified organisms, suggest that regular dental maintenance may increase the likelihood of PJI compared to patients without teeth.
